# Safety and efficacy of minimally invasive McKeown esophagectomy in 1023 consecutive esophageal cancer patients: a single-center experience

**DOI:** 10.1186/s13019-022-01781-2

**Published:** 2022-03-15

**Authors:** Xiao-Dong Zheng, Shi-Cong Li, Chao Lu, Wei-Ming Zhang, Jian-Bin Hou, Ke-Feng Shi, Peng Zhang

**Affiliations:** 1 Department of Cardiothoracic Surgery, Tianjin Medical University General Hospital, AnShan Road No. 154, Heping District, Tianjin, 30052 China; 2 Department of Thoracic Surgery, Anyang Tumor Hospital, The Fourth Affiliated Hospital of Henan University of Science and Technology, HuanBin North Road, No. 1, Anyang, 455000 Henan China

**Keywords:** Esophageal cancer, Cancer treatment, Minimally invasive esophagectomy, Complication, Prognosis

## Abstract

**Objective:**

By analyzing the perioperative, postoperative complications and long-term overall survival time, we summarized the 8-year experience of minimally invasive McKeown esophagectomy for esophageal cancer in a single medical center.

**Methods:**

This retrospective follow-up study included 1023 consecutive patients with esophageal cancer who underwent MIE-McKeown between Mar 2013 and Oct 2020. Relevant variables were collected and evaluated. Overall survival (OS) and disease-free survival (DFS) were analyzed by Kaplan–Meier method.

**Results:**

For 1023 esophageal cancer undergoing MIE-McKeown, the main intraoperative complications were bleeding (3.0%, 31/1023) and tracheal injury (1.7%, 17/1023). There was no death occurred during operation. The conversion rate of thoracoscopy to thoracotomy was 2.2% (22/1023), and laparoscopy to laparotomy was 0.3% (3/1023). The postoperative morbidity of complications was 36.2% (370/1023), of which anastomotic leakage 7.7% (79/1023), pulmonary complication 13.4% (137/1023), chylothorax 2.3% (24/1023), and recurrent laryngeal nerve injury 8.8% (90/1023). The radical resection rate (R0) was 96.0% (982/1023), 30-day mortality was 0.3% (3/1023). For 1000 cases with squamous cell carcinoma, the estimated 3-year and 5-year overall survival was 37.2% and 17.8% respectively. In addition, neoadjuvant chemotherapy offered 3-year disease-free survival rate advantage in advanced stage patients (for stage IV: 7.2% vs. 1.8%).

**Conclusions:**

This retrospective single center study demonstrates that MIE-McKeown procedure is feasible and safe with low perioperative and postoperative complications’ morbidity, and acceptable long-term oncologic results.

## Introduction

Esophageal cancer ranks the seventh common malignant tumor and the sixth leading cause of tumor-related death worldwide in 2018. In China, it is the sixth most frequently diagnosed cancer and the fourth leading cause of cancer death [1]. Despite great advances have been made in its diagnosis, surgical treatment and neoadjuvant therapy, probably due to its aggressiveness, the overall 5-year survival rate rests around 30% [2, 3]. Esophagectomy is an important, potentially curative treatment for resectable esophageal cancer. However, the conventional open esophagectomy is a technically challenging procedure, which is associated with significant complications’ morbidity and mortality [4, 5]. Therefore, minimally invasive esophagectomy (MIE) has been adopted worldwide with lower invasiveness.

Currently, MIE is commonly performed using two approaches: the MIE-McKeown and MIE Ivor-Lewis. MIE-McKeown with three-field lymph node dissection, which may obtain better survival than the two-field lymph node dissection, has evolved as one standard procedure of resectable esophageal cancer [6, 7]. However, there is no definitive scientific evidence supporting that the use of MIE as an alternative to open esophagectomy has advantages of significantly lowering morbidity of complications and mortality [8–10]. Moreover, the oncologic outcomes after minimally invasive surgery are still controversial [11–14].

In this study, we retrospectively analyzed 1023 consecutive patients with esophageal cancer undergoing MIE-McKeown in our hospital from Mar 2013 to Oct 2020. The aim of this paper was to evaluate the technical feasibility, safety and the clinical outcomes of MIE-McKeown in a large cohort of patients from Henan province, the highest incidence area of esophageal cancer in china.

## Methods

### Study design and participants

From Mar 2013 to Oct 2020, overall 1023 patients were enrolled, including 605 males and 408 females, with a mean age of 64.14 ± 7.12 years (range 39–85 years) and mean tumor length of 3.78 ± 1.57 cm (range 0.5–12 cm). For histopathology, the proportion of squamous cell carcinoma was 97.8% (1000/1023), small cell carcinoma with 1.4% (15/1023), carcinosarcoma with 0.4% (4/1023), and adenocarcinoma with 0.4% (4/1023). Other detailed clinical characteristics were presented in Table 1.

**Table 1:** Demographics and clinicopathological parameters (n = 1023)

Variables	N = 1023	Rate (%)
Demography	Age (mean ± SD)	64.14 ± 7.12
	Male: % (n/n)	59.2 (605/1023)
	Female: % (n/n)	40.9 (418/1023)
ASA-score	ASA-1: % (n/n)	70.0 (716/1023)
	ASA-2: % (n/n)	26.9 (275/1023)
	ASA-3: % (n/n)	3.1 (32/1023)
Comorbidity	Hypertension: % (n/n)	20.8 (213/1023)
	Diabetes: % (n/n)	6.6 (68/1023)
	COPD: % (n/n)	1.3 (13/1023)
	Liver cirrhosis: % (n/n)	0.7 (7/1023)
	Coronary artery disease:% (n/n)	2.5 (26/1023)
Location of lesion	Upper third: % (n/n)	23.2 (238/1023)
	Middle third: % (n/n)	65.7 (672/1023)
	Lower third: % (n/n)	11.0 (113/1023)
Histological type	Squamous carcinoma: % (n/n)	97.8 (1000/1023)
	Adenocarcinoma: % (n/n)	0.4 (4/1023)
	Small cell carcinoma: % (n/n)	1.5 (15/1023)
	Sarcocarcinoma: % (n/n)	0.4 (4/1023)
Tumor size	≤ 3 cm: % (n/n)	40.1 (410/1023)
	3–5 cm: % (n/n)	35.1 (359/1023)
	≥ 5 cm: % (n/n)	24.8 (254/1023)
Surgery-alone	n (%)	91.1 (932/1023)
Pathological T stage (%)	Tis∼1: % (n/n)	23.6 (220/932)
	T2: % (n/n)	22.5 (210/932)
	T3: % (n/n)	44.9 (418/932)
	T4: % (n/n)	9.0 (84/932)
Pathological N stage (%)	N0: % (n/n)	61.3 (571/932)
	N1: % (n/n)	24.9 (232/932)
	N2: % (n/n)	10.8 (101/932)
	N3: % (n/n)	3.0 (28/932)
Pathological TNM stage (%)	I0-IB: % (n/n)	21.4 (199/932)
	IIA: % (n/n)	16.5 (154/932)
	IIB: % (n/n)	21.6 (201/932)
	IIIA: % (n/n)	6.3 (59/932)
	IIIB: % (n/n)	27.7 (258/932)
	IVA: % (n/n)	6.5 (61/932)
Neoadjuvant chemotherapy with surgery	% (n/n)	8.9 (91/1023)
	cIIIB: % (n/n)	70.3 (64/91)
	cIVA: % (n/n)	29.7 (27/91)
Thoracoscopy combined with laparotomy	% (n/n)	78.6 (804/1023)
Thoracoscopy combined with laparoscopy	% (n/n)	21.4 (219/1023)

All patients were diagnosed as esophageal cancer by upper gastrointestinal endoscopy and biopsy. Simultaneously, comprehensive pre-operative evaluations were carried out. Patients with pre-operative TNM stage (AJCC staging manual, 8th edition) > IIIa were recommened for neoadjuvant therapy. Meanwhile, patients with staging pIII-pIVA received adjuvant therapy.

The inclusion criteria were: (i) definitive diagnosis of esophageal cancer. (ii) preoperative clinical staging (AJCC.2018) T:1–4A, N:0–2, M:0; (iii) accepted MIE-McKeown. (iv) ASA score (American Society of Anesthesiologists) 1–3. The exclusion criteria were: (i) extensive thoracic or abdominal adhesion; (ii) patients with unresectable or distant metastasis; (iii) patients can not tolerate MIE for obvious dysfunction of vital organs; (iv) impaired coagulation; (v). death unrelated to the carcinoma.

### Study endpoints

The primary endpoint was the 3, 5-year overall survival (OS) time and progression-free survival (PFS). Secondary endpoints were the short-term outcomes, including operative time, total blood loss, R0 resection rate, total and positive numbers of dissected lymph nodes, 30-day postoperative mortality, length of hospital stay and ICU stay, postoperative recovery, and the incidence of treatment-related complications.

### Postoperative care

The patients were given enteral nutrition at postoperative 1-day through a nasal feeding tube, and started eating at 5–7 days after operation if there weren’t any signs of leakage and functional gastric conduit evacuating disturbance examined by a barium swallow on all patients.

### Follow-up

All patients received follow-up after operation, every 3-month during the first year and every 6-month thereafter at the outpatient department. Simultaneously, telephone follow-up every 3-month conducted by LinkDoc company. Overall survival (OS) and disease-free survival time was the interval from the day of surgery to death or the last follow-up date (Oct. 2019).

### Statistical analysis

Descriptive statistics were provided, continuous variables were presented as mean ± SD, categorical variables as distribution ratio. Survival was analyzed by Kaplan–Meier method for patients with squamous cell carcinoma. All statistical analyses were performed with a dedicated analysis tool (SPSS 25.0 statistical software package; SPSS, Chicago, Il, USA).

## Results

### Intraoperative complications and outcomes

A total of 1023 patients underwent MIE-Mckeown surgery successfully and without intraoperative death occoured. The surgical procedures and results were listed in Table 2. The most common severe intraoperative complications were hemorrhage (3.0%, 31/1023), and the next was tracheal injury (1.7%, 17/1023). 8 patients received emergency conversion to open surgery due to intraoperative bleeding (5 thoracotomy, 3 laparoscopy). Another 17 patients suffered from emergency conversion because of failing to one-lung ventilation (n = 2) and bulky tumor (n = 15).

**Table 2:** Operative and post-operative parameters

Variables	N = 1023	
Operation time (min)	Average (mean ± SD)	215.77 ± 50.33
Total blood loss (mL)	Average (mean ± SD)	224.13 ± 156.11
72 h-postoperative drain volume (ml)	Average (mean ± SD)	698.13 ± 428.34
Chest drain removal (day)	Average (mean ± SD)	2.02 ± 1.75
Post-operative hospital stay (days)	Average (mean ± SD)	16.69 ± 10.74
ICU stay (days)	Average (mean ± SD)	1.30 ± 2.62
No. of lymph nodes harvested	Average (mean ± SD)	19.09 ± 8.12
Mediastinal	Average (mean ± SD)	12.66 ± 5.97
Left recurrent laryngeal nerve	Average (mean ± SD)	2.35 ± 1.74
Right recurrent laryngeal nerve	Average (mean ± SD)	2.27 ± 1.98
Abdominal: average	Average (mean ± SD)	6.43 ± 4.79
No. stations of lymph node dissected	Average (mean ± SD)	6.63 ± 1.46
R0 resection	% (n/n)	96.0 (982/1023)
Intraoperative complication		
Bleeding	% (n/n)	3.0 (31/1023)
Tracheal injury	% (n/n)	1.7 (17/1023)
Conversion thoracoscopy	% (n/n)	2.2 (22/1023)
Reason for conversion		
Failure of lung septation	Constituent ratio % (n/n)	9.1 (2/22)
Bleeding	Constituent ratio % (n/n)	22.7 (5/22)
Bulky tumor (T4)	Constituent ratio % (n/n)	68.2 (15/22)
Pleural or peritoneal adhesions	Constituent ratio % (n/n)	0
Conversion laparoscopy	% (n/n)	0.3 (3/1023)
Reason for conversion		
Bleeding	Constituent ratio % (n/n)	100 (3/3)

The average overall operation time was 215.77 ± 50.33 min (range 50–720 min) and the average intraoperative blood loss was 224.13 ± 156.11 ml (range 50–2300 ml). Postoperative thoracic tube drainage was performed for 1–22 days, with an average of 2.02 ± 1.75 days. The 72-h postoperative chest drainage volume was 698.13 ± 428.34 ml (range 30–3915 ml). The average post-operative hospital stay was 16.69 ± 10.74 days (range 7–86 days).

The median mean number of lymph nodes retrieved was 19.09 ± 8.12 (range 6–58), and the harvested lymph node station was 6.63 ± 1.46. The average number of harvested mediastinal lymph nodes was 12.66 ± 5.97 (range 2–50), and the harvested abdominal nodes was 6.43 ± 4.79 (range 0–33). The radical resection (R0) rate was 96.0% (982/1023).

### Postoperative complications

Detailed post-operative complications were shown in Table 3. Major surgical complications occurred in 370 patients (36.2%, 370/1023). Unplanned secondary operation was performed in 10 cases. Vocal cord palsy developed in 90 patients (8.8%) in whom 68 cases recovered within 3–6 weeks, 22 patients’ vocal cord palsy was permanent. Anastomotic leakage was detected in 79 patients (7.7%) in whom 2 cases required a follow-up operation, 1 cases eventually died of tracheal fistula, and the remaining cases were managed conservatively through nutritional support. 24 cases (2.3%) diagnosed with chylothorax in whom 4 cases received secondary operation (thoracic duct ligation), and the remaining cases were managed conservatively. 53 (5.2%) cases were diagnosed as anastomotic stenosis and cured by gastroscopic dilatation or esophageal stenting. The most common non-surgical complications was pulmonary complications (n = 137), in whom 94 cases with respiratory pneumonia, 18 with ARDS, 12 with pneumothorax, 10 with respiratory failure, and 3 with pulmonary embolism. 45 (4.4%) cases developed atrial fibrillation, and 14 (1.4%) cases developed delayed gastric emptying, of which 2 cases were treated with conservative treatments such as pyloric stents.

**Table 3:** Postoperative complications

Variables	N	% (n/n)	Constituent ratio % (n/n)
Total post-operative complications	370	36.2 (370/1023)	–
Major surgical complications			
Unplanned second operation	10	1.0 (10/1023)	
Post-operative hemorrhage	4	0.4 (4/1023)	
Chyle leakage	4	0.4 (4/1023)	
Anastomotic leak	2	0.2 (2/1023)	
Anastomotic leakage	79	7.7 (79/1023)	
Type I (conservative)	18		22.8 (18/79)
Type II (nonsurgical intervation)	59		74.7 (59/79)
Type III (second operation)	2		2.5 (2/79)
Anastomotic stenosis	53	5.2 (53/1023)	
Vocal cord palsy	90	8.8 (90/1023)	
Temporary (recovered in 2 weeks)	68		75.6 (68/90)
Permanent	22		24.4 (22/90)
Chylothorax	24	2.3 (24/1023)	
Type I (Low fat dietary)	5		20.8 (5/24)
Type II (total parenteralnutrition)	15		62.5 (15/24)
Type III (surgical intervation)	4		16.7 (4/24)
Major non-surgical morbidity			
Pulmonary complication	137	13.4 (137/1023)	
Respiratory pneumonia	94		68.6 (94/137)
ARDS	18		13.1 (18/137)
Pneumothorax	12		8.8 (12/137)
Respiratory failure	10		7.3 (10/137)
Pulmonary embolism	3		2.2 (3/137)
Atrial fibrillation	45	4.4 (45/1023)	
Delayed gastric emptying	14	1.4 (14/1023)	
30-Day mortality	3	0.3 (3/1023)	
Anastomotic leak	1	0.1 (1/1023)	33.3 (1/3)
DIC	1	0.1 (1/1023)	33.3 (1/3)
Aspiration pneumonia	1	0.1 (1/1023)	33.3 (1/3)

The 30-day mortality rate was 0.3% (3/1023) and the causes of death were pulmonary infection (33.3%, 1/3), anastomotic leakage (33.3% 1/3) and DIC (33.3% 1/3) respectively.

### Follow-up and survival analysis

**Fig. 1:**
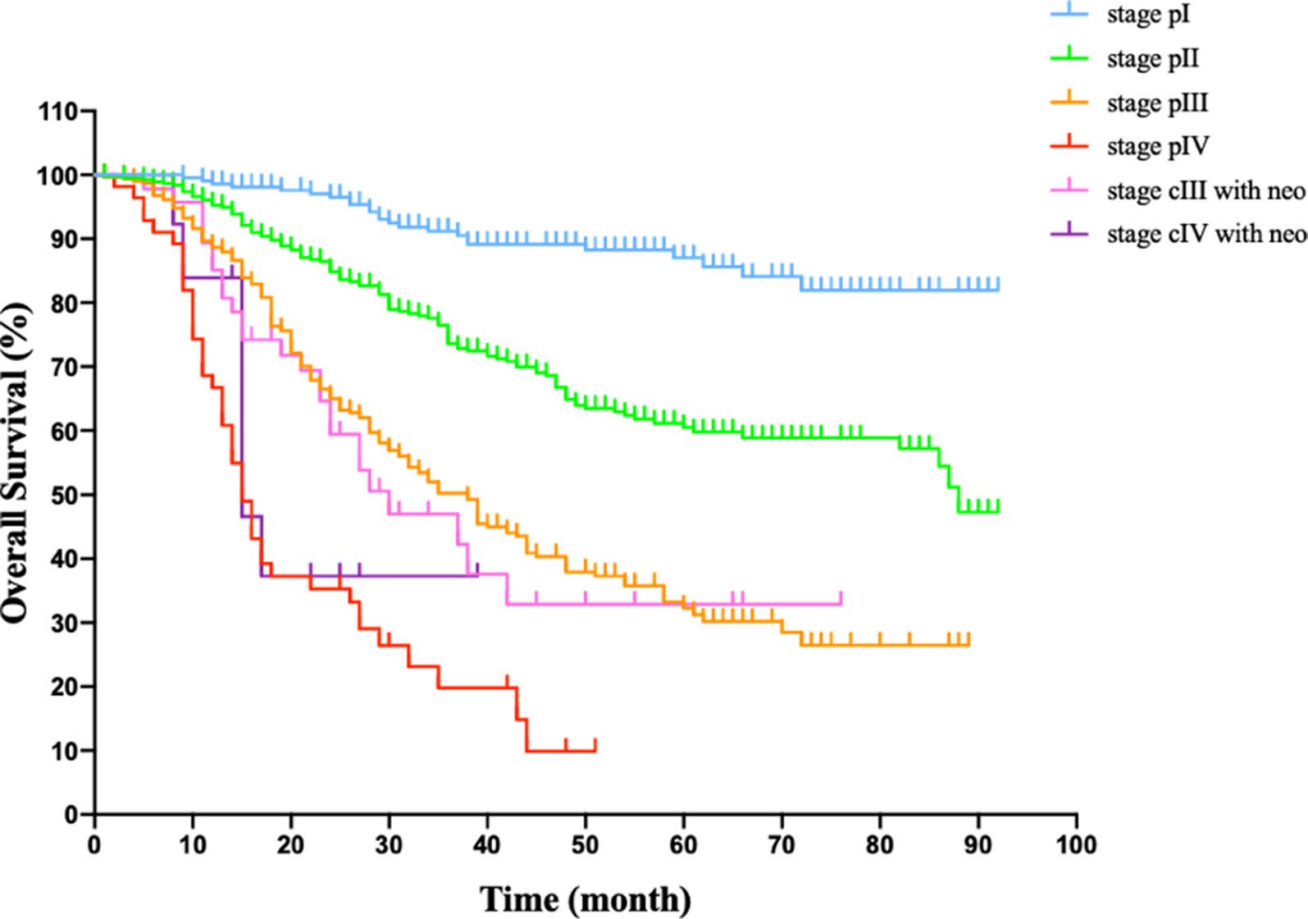
Kaplan–Meier survival curve of patients presenting with esophageal cell carcinoma who received the minimally invasive McKeown esophagectomy, stratified by stage

For 1023 cases with squamous cell carcinoma, successful follow-up was completed on 1023 patients (100%), up to the last follow up in Oct. 2020. The median follow-up time was 32 months (range 1–92 months). The estimated 3-year overall survival rate was 37.2%, the estimated 5-year overall survival rate was 17.8%. In the subsets of stage I, II, III without neoadjuvant therapy, IV without neoadjuvant therapy, the estimated 3-year overall survival rate were 60.7%, 44.1%, 44.2%, 7.1% respectively. In the subsets of stage I, II, III without neoadjuvant therapy, the estimated 5-year overall survival rate were 30.8%, 21.7%, 9.7% respectively. For patients undergoing neoadjuvant therapy, the estimated 3-year overall survival rate were 14.9% and 6.4% for stage cIII and stage cIV, the estimated 5-year overall survival rate was 6.4% for stage cIII. Neoadjuvant therapy have not been found to significantly improve the overall survival rate in advanced stage patients. Kaplan–Meier survival curve among each TNM stage was plotted and demonstrated in Fig. 1.

**Fig. 2:**
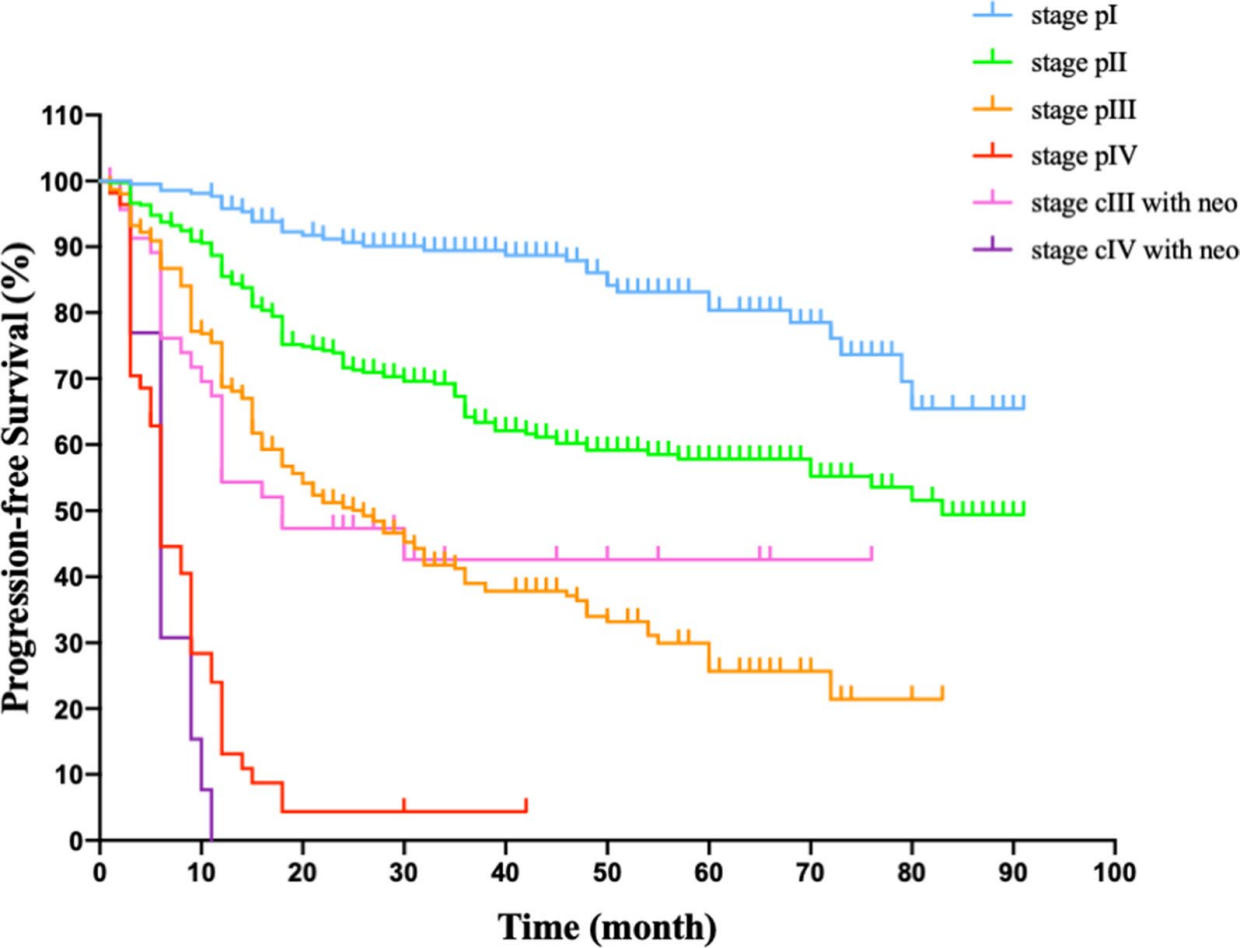
Kaplan–Meier survival curve of patients presenting with esophageal cell carcinoma who received the minimally invasive McKeown esophagectomy, stratified by stage

For 1023 cases with squamous cell carcinoma, successful follow-up was completed on 1023 patients (100%), up to the last follow up in Oct. 2020. The median disease-free survival was 32 months (range 1–92 months). The estimated 3-year median disease-free survival rate was 32.5%, the estimated 5-year median disease-free survival rate was 14.3%. In the subsets of stage I, II, III without neo-adjuvant therapy, IV without neo-adjuvant therapy, the estimated 3-year median progression-free survival were 56.1%, 38.9%, 18.1%, 1.8% respectively. In the subsets of stage I, II, III without neoadjuvant therapy, the estimated 5-year disease-free rate were 25.7%, 17.8%, 6.5% respectively. For patients undergoing neoadjuvant therapy, the estimated 3-year median progression-free survival rate were 12.8% for stage cIII and 7.2% for cIV, the estimated 5-year disease-free survival rate were 6.4% for stage cIII. In the group of stage IV, neoadjuvant therapy offered advantages in disease-free survival rate (7.2% vs. 1.8%). Kaplan–Meier survival curve among each TNM stage was plotted and demonstrated in Fig. 2.

## Discussion

Esophagectomy and reconstruction remain the standard procedure in the curative intent treatment for patients with resectable esophageal cancer. In attempts to reduce the complications’ morbidity and mortality of open esophagectomy, MIE has been widely used worldwide in recent years. Some prospective and meta-analysis studies have showed the potential short-term benefits of MIE, including less blood loss, lower incidence of respiratory complications, shorter hospital or ICU stays, and faster surgical recovery [13, 15–17]. Currently, McKeown with cervical esophagogastric anastomosis and lvor Lewis with thoracic esophagogastric anastomosis surgery were the most common surgical procedures in esophagectomy. Several studies have showed that MIE-McKeown procedure was associated with higher incidence of recurrent laryngeal nerve injury, pneumonia and anastomotic leakage than Lvor–Lewis [18–21]. However, MIE-McKeown has some potential benefits, such as more proximal resection margin and more lymph nodes dissected, which seems to improve long-term survival [22, 23]. Thus, the MIE-McKeown procedure combined three-field lympadenectomy is recommended as a routine approach in China and Japan [7, 23, 24]. At present, approximately 700 patients with esophageal cancer underwent MIE-McKeown at our institution per year. This study included 1023 consecutive cases undergoing MIE-McKeown procedure, representing a single center large sample study, suggesting that the MIE-McKeown is feasible and can be safely performed with a 30-day mortality rate of 0.3%, and a median postoperative hospital stay of 12 days. In term of main postoperative complications, anastomotic leakage rate 7.7%, pulmonary complication rate 13.4%, chyle leakge rate 2.3% and recurrent laryngeal nerve (RLN) injury rate 8.8%. These short-term outcomes of the procedure at our institution are equivalent to or superior to most published literatures [21, 25–28].

According to our experience, prevention is key to the treatment of complications. Bleeding is known to be a common cause of 2 exploration after esophagectomy, accounting for 40% (4/10) of all secondary operation in our study. Hemorrhages and incision bleeding can be effectively prevented by careful examinations. In addition, chyle leakage is another common complication in open esophagectomy or MIE, causing secondary surgical exploration. The previously reported incidence varies from 0.8 to 5.9% [25, 29] and 2.3% in our study. At present, the effectiveness of prophylactic thoracic duct ligation on preventing chylothorax still remains controversial [29, 30]. Moreover, the potential impact of thoracic duct ligation on long-term survival has not been determined. According to our experience, routine thoracic duct ligation was recommended for middle or upper esophageal cancer with T3–T4, and should be performed when thoracic duct was damaged definitely or suspiciously. In addition, several studies found that preoperative oral administration of olive oil can effectively pevent chylothorax [31, 32].

Anastomotic leakage after esophagectomy is one of the most severe complications. Some studies have shown that the incidence of neck anastomosis in Mckeown surgery is higher than that in Ivor Lewis surgery with intrathoracic anastomosis [18–20]. The reported incidence varies from 5 to 23.3% [15, 18, 21, 25, 27, 33] and 7.7% in our study. Tracheal fistula, one of the most fatal complications, accounting for 33.3% (1/3) of all deaths within 30 days after surgery in our study, was often caused by gastric acid or other pollutant secretions from anastomotic leakage. A large sample review, including 25 articles, gave evidence that anemia, increased amount of blood loss, low pH and high PCO_2_ values, prolonged surgery time and poor technique independently increased the risk of anastomotic leakage [34]. Therefore, some measures, such as gastric protection of blood supply, improvement of anastomotic technique, perioperative nutrition support and relieving the tension of anastomosis, can effectively reduce the incidence of the complications [34–36].

Besides anastomotic leak, pneumonia was the most observed complication following MIE. Previous studies showed that its incidence ranged from 9.0 to 31.9%, and was 13.4% in our study. Most of these cases were cured by conservative treatment and with 1 case died of aspiration pneumonia, which caused by recurrent laryngeal nerve (RLN) injury. Notably, RLN injury can be caused by contusions, excessive stretching and thermal damage occurring when dissecting the lymph nodes surrounding the left and right RLN [38]. So, it is essential to protect RLN from burning or shearing during manipulation.

Considering the high incidence of lymphatic metastasis surrounding RLN [39], we must dissect the lymph nodes in these fields. However, the meticulous dissection in a narrow space is still challenging and frequently leads to RLN palsy [37, 38]. As operative experience increased, the rate of hoarseness in our center gradually decreased by avoiding stretching, compression and thermal injury on the RLN. Regarding the left RLN prevention, it is critical to clearly expose the RLN bluntly before lymph node dissection. In order to obtain an optimal visualization, we used some methods, such as the single lumen intubation, turn lateral decubitus position to semi prone position, assistant’ pull force from opposite side, which were conducive to dissect the left RLN easily [40].

The potential benefits of MIE-McKeown are a more proximal resection margin and improved lymph node dissection, which can provide more accurate pathological staging and improve patient survival, especially for patients with middle or upper thoracic esophageal cancer [7, 22, 23]. However, several prospective and observed studies showed that MIE-McKeown with three-filed lymphadenectomy has not prolonged the survival time than open procedure or MIE lvor-Lewis, and even though yielded more lymph nodes [41–44]. In our study, 55.9% of cases were in stage of T3–T4, 38.7% with nodal positive (N1–3), and 34.2% with locally advanced esophageal carcinoma (IIIB + IVA stage). Our results showed that MIE-McKeown provided a high percentage of R0 radical resections (96.0%), adequate lymphadenectomy (the mean number of lymph nodes dissected 19.09, the mean number of lymph node stations 6.63), and obtained a 37.2% of 3-year OS rate and 17.8% of 5-year OS rate in squamous cell carcinoma, which was equivalent or superior to that reported for open esophagectomy or MIE Lvor-Lewis [12, 14, 23, 27, 28, 34, 36]. These results indicated that MIE McKeown method satisfied the oncological requirements and did not impact long-term survival rates. In addition, neoadjuvant chemotherapy seemed to offer 3-year disease-free survival advantage in advanced stage patients (for stage IV: 7.2% vs. 1.8%). Mantel-Cos test was conducted to analyze the 3-year disease-free survival and suggests that p-vales exceeds 0.05 (p-vales = 0.832). While neoadjuvant chemotherapy offers no significant advantage of overall survival and disease-free survival in other patients. Given the advanced patients who accepted neoadjuvant chemotherapy at least detriment in physical and nutritional status, hampering the ability to deliver adjuvant treatment.

Recent guidelines about esophageal squamous cell carcinoma (SCC) recommend the use of multimodal treatment (pre-operative chemo-radiation therapy) for cT1b-T4a or upfront esophagectomy for cT1b-T2N0 patients. However, in our study, the use of multimodal treatment is low. On the one hand, patients have high acceptance of surgery and low acceptance of neoadjuvant therapy. In addition, neoadjuvant chemotherapy cannot solve the problem of swallowing obstruction in locally advanced esophageal cancer in the shortest time, causing patients to actively refused neoadjuvant therapy. In future medical activities, we should strengthen the education of neoadjuvant therapy to patients, so that they will understand the long-term advantages of neoadjuvant therapy, so as to increase the probability of neoadjuvant therapy.

However, this clinical research has a number of limitations. First, this trial was a single central retrospective study lacking of control group to compare the surgical outcomes. Second, data on quality of life, functional results and cost-effectiveness have not been studied. Third, in this study, neoadjuvant therapy followed by esophagectomy for locally advanced ESCC seemed to improve long-term survival compared with surgery alone, but we did not conduct a rigorous design to confirm the survival advantage. Lastly, although the sample size is large in this study, multicenter randomized controlled trials (RCTs) should be conducted in the future work to verify the equivalency or advantages of MIE-Mckeown compared with open esophagectomy or MIE-Lvor Lewis.

## Conclusion

This single-center experience demonstrates that MIE-McKeown with standard three-field lymphadenectomy is a safe and feasible procedure of resectable esophageal cancer. It offers satisfactory surgical outcomes and long-term clinical outcomes such as a high radical resection rate, adequate lymphadenectomy, and long overall survival time. Further researches with prospective multicenter randomized controlled trials are needed to clinically validate these findings.

## Data Availability

The data that support the findings of this study are available from the corresponding author upon reasonable request.

## Abbreviations

OS: Overall survival
DFS: Disease-free survival
MIE: Minimally invasive esophagectomy
RLN: Recurrent laryngeal nerve
RCTs: Randomized controlled trials
